# Asymptomatic myocardial ischemia forecasts adverse events in cardiovascular magnetic resonance dobutamine stress testing of high-risk middle-aged and elderly individuals

**DOI:** 10.1186/s12968-018-0492-5

**Published:** 2018-11-22

**Authors:** R. Brandon Stacey, Trinity Vera, Timothy M. Morgan, Jennifer H. Jordan, Matthew C. Whitlock, Michael E. Hall, Sujethra Vasu, Craig Hamilton, Dalane W. Kitzman, W. Gregory Hundley

**Affiliations:** 1 Department of Internal Medicine, Cardiovascular Medicine Section, Wake Forest School of Medicine, Medical Center Boulevard, Winston-Salem, North Carolina 27157-1045 USA; 2 Department of Public Health Sciences, Wake Forest School of Medicine, Winston-Salem, NC USA; 3 Department of Radiology (Division of Radiologic Sciences), Wake Forest School of Medicine, Winston-Salem, NC USA; 4 Department of Medicine (Cardiovascular Medicine), University of Mississippi Medical Center, Jackson, MS USA; 5 Department of Medicine (Cardiovascular Medicine), Stanford University School of Medicine, Palo Alto, CA USA

**Keywords:** Stress testing, Cardiovascular events, Aging, Sex difference, Cardiovascular magnetic resonance

## Abstract

**Background:**

Current guidelines for assessing the risk of experiencing a hospitalized cardiovascular (CV) event discourage stress testing of asymptomatic individuals; however, these recommendations are based on evidence gathered primarily from those aged < 60 years, and do not address the possibility of unrecognized “silent myocardial ischemia” in middle aged and older adults.

**Methods:**

We performed dobutamine cardiovascular magnetic resonance (CMR) stress testing in 327 consecutively recruited participants aged > 55 years without CV-related symptoms nor known coronary artery disease, but otherwise at increased risk for a future CV event due to pre-existing hypertension or diabetes mellitus for at least 5 years. After adjusting for the demographics and CV risk factors, log-rank test and Cox proportional hazards models determined the additional predictive value of the stress test results for forecasting hospitalized CV events/survival. Either stress-induced LV wall motion abnormalities or perfusion defects were used to indicate myocardial ischemia.

**Results:**

Participants averaged 68 ± 8 years in age; 39% men, 75% Caucasian. There were 38 hospitalized CV events or deaths which occurred during a mean follow-up of 58 months. Using Kaplan-Meier analyses, myocardial ischemia identified future CV events/survival (*p* <  0.001), but this finding was more evident in men (p <  0.001) versus women (*p* = 0.27). The crude hazard ratio (HR) of myocardial ischemia for CV events/survival was 3.13 (95% CI: 1.64–5.93; *p* < 0.001). After accounting for baseline demographics, CV risk factors, and left ventricular ejection fraction/mass, myocardial ischemia continued to be associated with CV events/survival [HR: 4.07 (95% CI: 1.95–8.73) *p* < 0.001].

**Conclusions:**

Among asymptomatic middle-aged individuals with risk factors for a sentinel CV event, the presence of myocardial ischemia during dobutamine CMR testing forecasted a future hospitalized CV event or death. Further studies are needed in middle aged and older individuals to more accurately characterize the prevalence, significance, and management of asymptomatic myocardial ischemia.

**Trial registration:**

(ClinicalTrials.gov identifier): NCT00542503 and was retrospectively registered on October 11th, 2007.

## Background

Whether to assess or how best to manage silent myocardial ischemia is not well defined. In a general asymptomatic population, the prevalence of silent myocardial ischemia is estimated to be between 2 and 5%, and in those with a prior myocardial infarction (MI), silent myocardial ischemia may be as high as 30% [1]. Identification of silent myocardial ischemia may be obtained with dobutamine stress testing [2–5]. Individuals with silent myocardial ischemia have the same level or higher risk for cardiovascular events and mortality as patients who present with typical angina [6–9].

To identify those at risk of a future cardiovascular (CV) event, current guidelines from the American College of Cardiology (ACC), the American Heart Association (AHA), and the European Society of Cardiology (ESC) recommend against stress testing in individuals who do not exhibit anginal symptoms consistent with CV disease. [10, 11] ACC Appropriateness Use Criteria regard the utility of stress testing asymptomatic individuals with multiple risk factors for a CV event as “uncertain” [12–15]. Therefore, when following current guidelines, one often does not perform stress testing to identify silent myocardial ischemia unless patients exhibit symptoms that may relate to angina.

Interestingly, many of these recommendations rely on study results involving younger (aged 35 to 60 years) who were relatively active individuals with a low prevalence of “silent ischemia.” In a retrospective review of nearly 2000 exercise stress echocardiograms, inducible ischemia was not associated with death, but nearly half of the study population was younger than 50 years [16]. A different study in patients aged 50–75 years which included over 600 relatively healthy patients found a three-fold increase in the risk of CV events in those who had silent myocardial ischemia [17]. Older individuals, who may be less active than their younger counterparts, may not develop symptoms, and thus, it remains uncertain as to whether current AHA/ACC appropriateness criteria are arranged to identify silent myocardial ischemia in the elderly.

Accordingly, we hypothesized that silent myocardial ischemia is present in higher-risk middle-aged and elderly individuals, and its presence would identify those at higher risk for CV events and death during follow-up after accounting for the presence of traditional CV disease risk factors. This prospective study funded by combined resources of the National Heart Lung and Blood Institute and the National Aging Institute of the National Institutes of Health within the United States was performed to address a gap in knowledge related to the utility of CV stress testing in middle-aged and older individuals with a) risk factors for a CV event (evaluated via calculation of their Framingham risk score), b) no concurrent symptoms associated with CV disease, and c) the potential presence of unrecognized silent ischemia.

## Methods

### Study design

The study was approved by the Institutional Review Board of Wake Forest Health Sciences, and each participant provided witnessed, written informed consent. This study was registered with Clinicaltrials.gov (NCT00542503) and funded by National Institutes of Health grants R01HL076438 and P30AG21332. The purpose of this joint initiative was to the utility of pharmacologic cardiovascular magnetic resonance (CMR) stress testing results to identify those at risk of future hospitalizations for cardiac events. Upon enrollment risk factors for cardiac events, vital signs and fasting blood samples were collected; thereafter, each participant underwent a dobutamine stress CMR (DCMR) test in which hemodynamic and left ventricular [LV] volumes, mass, ejection fraction and stress induced LV wall motion abnormalities were recorded.

After stress testing, active surveillance for hospitalized cardiac events was performed through follow-up telephone interviews conducted at 4-month intervals by a research nurse who was blinded to the DCMR results. If an event was suspected during the phone interview, it was substantiated by thorough review of the participant’s medical record. Clinical hospitalization events included a) incident heart failure (defined as the acute onset of dyspnea, chest x-ray evidence of congestion or a serum B-type natriuretic peptide level > 100 pg/ml, and receipt of intravenous diuretics), b) myocardial infarction (angina of ≥20 min duration and a rise in troponin or creatine kinase level above the 99 percentile of the upper reference limit) [18], c) unstable angina warranting coronary artery revascularization, d) sudden cardiac death (death during the hospital admission for acute coronary syndrome, significant cardiac arrhythmia, refractory heart failure, or death at home after chest pain complaint), or e) transient ischemic attack or cerebrovascular accident. Any participants who experienced an epicardial coronary artery revascularization procedure within 6 weeks of DCMR were excluded from the longitudinal event analysis.

### Study population

The study included participants from central and western North Carolina who possessed established risk factors (hypertension, diabetes) for a future hospitalized cardiac event for more than 5 years prior to study enrollment. This 5-year pre-requisite of a risk factor was suggested by NHLBI to address concerns of increasing risk suspected for individuals with longstanding CV disease. Potential participants were excluded if a) they had known coronary artery disease (CAD) or had experienced a prior myocardial infarction, b) reported any cardiovascular related symptoms such as chest pain or shortness of breath at rest or with exertion 6 months prior to enrollment, or c) exhibited a contraindication to intravenous dobutamine or CMR exam (e.g., presence of incompatible bio-metallic implants or claustrophobia). Recruitment of study participants was achieved through newspaper and television advertisements and mailings to randomly selected individuals 55 to 90 years within the catchment area. To define certain covariates, such as hypertension and cholesterolemia, patients were categorized by JNC-7 and NCEP ATP-III, respectively, or by prior provider-based diagnosis [19, 20].

### DCMR stress test procedure

The DCMR stress test protocol was accomplished according to previously published techniques, [21–24] and images were acquired on a 1.5T (Avanto, Siemens Healthineers, Erlangen, Germany) whole-body imaging system. LV cines were obtained in multiple contiguous short axis slices (apex to base) and in 3 long axis views (2, 3, and 4 chamber) at baseline, peak dobutamine stress, and then after 10 min of recovery. To achieve peak stress, dobutamine was titrated up to 40 μg/kg/min (without or with up to 1.5 mg of atropine) to achieve 80% of the maximum predicted heart rate response for age. This target heart rate response was selected based on our prior studies demonstrating its efficacy for a) identifying inducible ischemia and b) adverse cardiac prognoses [22]. If the heart rate was more than 30 beats under the target heart rate at 20 μg/kg/min of dobutamine, atropine was administered. Brachial artery systolic (SBP) and diastolic blood pressure (DBP) were measured with an automatic CMR compatible sphygmomanometer.

### LV wall motion analysis

**Fig. 1:**
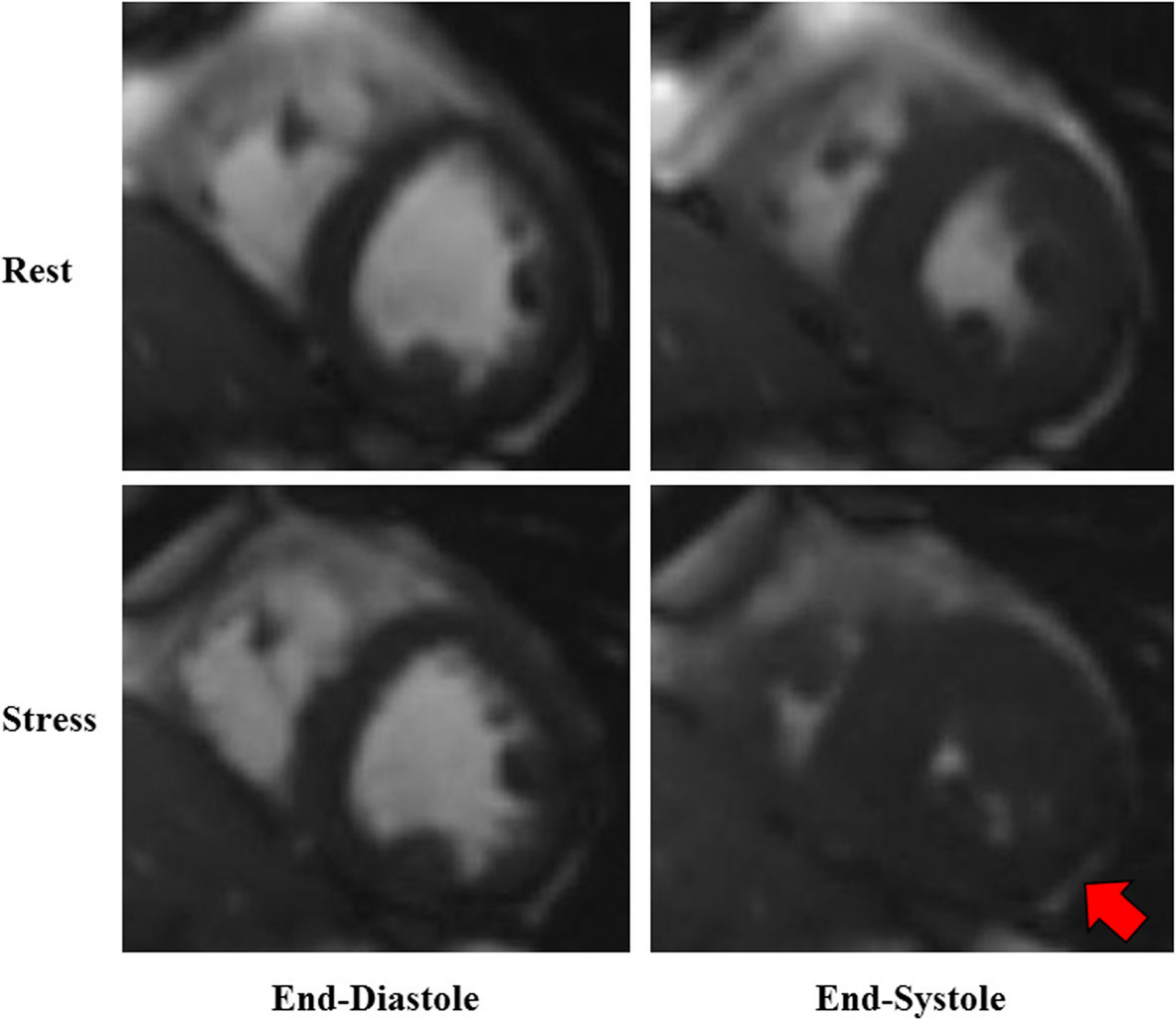
Dobutamine Stress-induced Wall Motion Abnormality. Stress-induced left ventricular (LV) wall motion abnormality obtained at peak DCMR. (Rest images on top; Stress images on bottom; Red arrow points toward an area of decreased myocardial thickening during stress)

The LV wall motion at baseline, peak dobutamine stress and in recovery was assessed with a visual scoring system in which 17 LV segments were scored according to AHA guidelines by CMR trained cardiologists (see Fig. 1) [22]. Inducible LV wall motion abnormalities were defined as an increase in a score of ≥ 1 (e.g., normal to hypokinetic) in 2 or more contiguous myocardial segments. Segments with an LV wall motion score of 2 or 3 at rest with no worsening of wall motion were considered negative for ischemia [24]. Also, per previously published techniques, LV volumes were measured from the short-axis series of cine white blood imaging sequences using a modified Simpson’s rule method [25]. Image acquisition parameters included a 45 msec repetition time (TR), a 1 msec echo time (TE), a 78° flip angle (FA), a 400 × 324 mm field of view (FOV), a 192 × 109 matrix, and an 8 mm thick slice with a 2 mm gap and an acceleration factor of 2.

### LV perfusion analysis

**Fig. 2:**
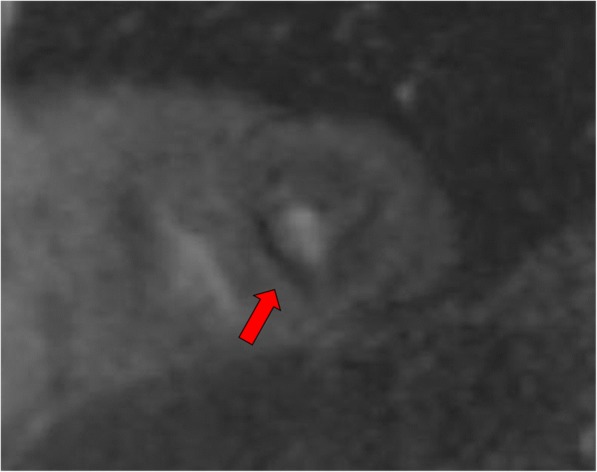
Stress-induced Myocardial Perfusion Defect: Apical stress-induced perfusion defect obtained at peak stress during dobutamine stress cardiovascular magnetic resonance (DCMR) stress test. Red arrows highlight lack of contrast relative to other myocardial segments to indicate a stress-induced perfusion defect

In those individuals with estimated glomerular filtration rates of > 60 ml/min., first pass perfusion imaging with gadobenate dimeglumine (0.1 mmol/kg; Multihance, Bracco Diagnostics Princeton, New Jersey, USA) was performed when 80% of the maximum predicted heart rate was achieved. At peak stress, 2 slices for assessing myocardial first pass perfusion were obtained. These perfusion images were collected in the short axis orientation in the middle and apical segments (2 slice positions due to the rapid heart rate). Image parameters included an 8 mm thick slice, TR 169 msec, TE 1.1 msec, FA of 12°, FOV of 360 × 270 mm and 192 × 108 matrix. Rest first-pass perfusion imaging was not performed. Any perfusion defect that persisted for more than 5 frames from onset of myocardial enhancement and encompassed > 25% of the thickness of the wall was further evaluated for classification as ischemic (see Fig. 2) [24].

### Myocardial ischemia

For the purposes of these analyses, unless otherwise specified, myocardial ischemia was defined according to previously published criteria including the presence of a stress-induced wall motion abnormality or the presence of a stress-induced perfusion defect for those who received contrast [24].

### Statistical analyses

Participants were analyzed in their entirety and also stratified by gender and the presence or absence of hospitalized CV events during the follow-up period. Fischer’s exact tests for dichotomous risk factor variables and two sample Student’s t-tests for continuous data were used to evaluate differences between those who did and did not experience hospitalized CV events. Cox proportional hazards regression models were used to determine the univariable association with each risk factor variable separately and the hazard of experiencing a hospitalized CV event. The increased or decreased risk of a future hospitalized CV event due to the presence or absence of a given variable was expressed by a hazard ratio (HR) with a corresponding 95% confidence interval (CI). A Cox multivariable model was constructed with a stepwise selection method using a *p*-value of 0.25 to enter or a p-value of 0.10 stay in the model to guard against over-fitting. Kaplan-Meier estimates were used to estimate event rates between those who did and did not demonstrate myocardial ischemia. These differences were also statistically evaluated using the log-rank test. Finally, multi-variate Cox proportional hazard models were used with incremental adjustment to evaluate the relationship between myocardial ischemia and clinical events. The different models used for this adjustment were as follows:

Model 1: age, race, gender, height, weight

Model 2: Model 1 + diabetes mellitus, hypertension, tobacco use, atrial fibrillation, hypercholesterolemia, systolic blood pressure

Model 3: Model 2 + left ventricular ejection fraction, left ventricular mass

All statistical analyses were performed with SAS JMP Pro 13.0 software package (SAS Institute, Cary, North Carolina, USA).

## Results

The age of the 327 participants within the study averaged 68 ± 8 (range 55 to 86) years; 39% men, 75% Caucasian, 22% African-American. The study population’s demographic data are displayed in Table 1. The pre-test likelihood for CAD was 30%. The imaging associated results are shown in Table 2. Of those included in our study identified as having myocardial ischemia, 19 (5.8%) had stress-induced wall motion abnormalities only, 38 (11.5%) had a stress-induced perfusion defect only, and 22 (6.7%) had both a stress-induced wall motion abnormality and a perfusion defect. Contrast was administered to 222 (67.9%) of all participants because 108 participants had an estimated glomerular filtration rate < 60 ml/min (a pre-determined threshold for which gadolinium contrast would not be administered).

**Table 1:** Baseline Characteristics of Demographics and Medical History by Events/Gender

	Women			Men		
	No CV events	CV events	*p*-value	No CV events	CV events	*p*-value
	(*n* = 177)	(*n* = 22)		(*n* = 112)	(*n* = 16)	
Age (years)	68.7 ± 8	69 ± 7	0.90	69 ± 8	72 ± 9	0.29
Caucasian (n, %)	130 (73%)	15 (68%)	0.36	86 (77%)	15 (94%)	0.34
African-American (n, %)	4 (24%)	6 (28%)	–	22 (20%)	1 (6%)	–
Body Mass Index (kg/m^2^)	31 ± 6	32 ± 6	0.26	29 ± 5	30 ± 7	0.53
Height (cm)	162 ± 7	164 ± 8	0.26	175 ± 8	175 ± 9	0.98
Weight (kg)	81 ± 18	89 ± 18	0.12	91 ± 17	95 ± 16	0.55
Total Cholesterol (mg/dL)	163 ± 46	172 ± 67	0.59	143 ± 36	151 ± 40	0.59
High Density Lipoprotein Cholesterol (mg/dL)	51 ± 14	49 ± 16	0.65	42 ± 14	41 ± 11	0.80
Hypertension, n (%)	168 (95%)	21 (95%)	0.91	102 (92%)	16 (100%)	0.09
Transient Ischemic Attack /Stroke, n (%)	6 (3%)	1 (5%)	0.76	9 (8%)	3 (18%)	0.21
Hypercholesterolemia, n (%)	128 (72%)	12 (55%)	0.09	68 (61%)	9 (57%)	0.73
Current smoker, n (%)	7 (4%)	2 (9%)	0.32	4 (4%)	1 (6%)	0.62
Diabetes, n (%)	64 (36%)	12 (54%)	0.09	50 (45%)	6 (38%)	0.59
Estimated Glomerular Filtration Rate (mL/min/1.73 m^2^)	58 ± 6	57 ± 9	0.58	58 ± 5	58 ± 4	0.99
Aspirin	109 (71%)	13 (62%)	0.6	84 (73%)	7 (53%)	0.41
Angiotensin Converting Enzyme Inhibitor	72 (41%)	6 (28%)	0.21	54 (48%)	8 (50%)	0.89
Beta Blocker	60 (35%)	7 (64%)	0.83	28 (25%)	3 (19%)	0.57
Diuretic	109 (62%)	13 (59%)	0.82	64 (57%)	9 (56%)	0.94
Angiotensin Receptor Blocker	65 (36%)	10 (45%)	0.43	27 (24%)	3 (18%)	0.62
Statin	129 (73%)	13 (59%)	0.19	82 (78%)	12 (75%)	0.78
Aldosterone antagonist	5 (3%)	1 (5%)	0.67	1 (1%)	0 (0%)	0.61
Calcium Channel Blocker	44 (25%)	9 (41%)	0.12	37 (33%)	6 (37%)	0.74

Baseline demographics and clinical characteristics stratified by gender. Mean ± Standard Deviation or number (percent). A *p*-value < 0.05 indicates statistical significance

**Table 2:** Baseline Characteristics of Stress Testing and Cardiac Imaging Measures by Events/Gender

	Women			Men		
	No CV events	CV events	*p*-value	No CV events	CV events	*p*-value
	(*n* = 177)	(*n* = 22)		(*n* = 112)	(*n* = 16)	
Resting Systolic Blood Pressure (mmHg)	141 ± 17	150 ± 25	0.11	140 ± 17	148 ± 16	0.17
Resting Diastolic Blood Pressure (mmHg)	77 ± 10	83 ± 11	0.16	82 ± 11	84 ± 10	0.61
Resting Heat rate (beats/minute)	67 ± 11	66 ± 13	0.80	63 ± 11	69 ± 11	0.13
Peak Systolic Blood Pressure (mmHg)	126 ± 23	126 ± 31	0.98	129 ± 15.8	126 ± 19.5	0.79
Peak Diastolic Blood Pressure (mmHg)	64.9 ± 13.2	63.4 ± 18.3	0.84	75 ± 17	76 ± 16	0.85
Peak stress Heat rate (beats/minute)	126 ± 14	117 ± 20	0.04	125 ± 17	124 ± 11	0.95
Rate pressure product (mmHg-bpm)	15,923 ± 3437	14,897 ± 4376	0.38	16,133 ± 3740	15,813 ± 2984	0.82
Left Ventricular Ejection Fraction (%)	66 ± 7	63 ± 9	0.23	61 ± 8	60 ± 11	0.55
Left Ventricular End Diastolic Volume (ml/m^2^)	59 ± 15	59 ± 17	0.96	64 ± 14	69 ± 15	0.13
Left Ventricular End Systolic Volume (ml/m^2^)	20 ± 7	23 ± 13	0.32	25 ± 10	30 ± 12	0.026
Left Ventricular Stroke Volume (ml/m^2)	38 ± 8	36 ± 7	0.73	39 ± 9	39 ± 9	0.97
Left Ventricular Mass (g/m^2^)	61 ± 11	66 ± 9	0.08	72 ± 14	80 ± 12	0.17
Left Ventricular Inducible Wall Motion Abnormality, n (%)	18 (10%)	2 (9%)	0.87	12 (10%)	9 (56%)	< 0.001
Left Ventricular Stress-induced Perfusion Defect (%; of those who received contrast)	31 (27%)	6 (37%)	0.40	15 (19%)	8 (62%)	0.001
Myocardial ischemia (Wall Motion or Perfusion; out of all participants without known CAD)	39 (22%)	7 (32%)	0.32	22 (20%)	11 (69%)	< 0.001

Baseline stress test and imaging characteristics stratified by gender. Mean ± Standard Deviation or number (percent). A *p*-value < 0.05 indicates statistical significance. *CV* cardiovascular

Relative to men, women required less total dobutamine (30 ± 143 versus 357 ± 176 μg/kg, *p* = 0.004) and atropine (0.42 ± 0.30 versus 0.57 ± 0.31 mg, *p* < 0.001) to achieve their target heart rate. The difference in the total atropine persisted after adjusting for weight (5.3 ± 3.7 versus 6.3 ± 3.9 μg/kg, in women versus men, *p* = 0.05). There was no difference in the peak rate-pressure product between men and women (16,101 vs 15,816 mmHg-bpm, respectively; *p* = 0.57). Over 94% of those included followed up for > 3 years, but in those with < 3 years follow-up, the participants (21 participants) tended to be older (71.9 ± 6 vs 68.1 ± 8 years; *p* = 0.04), were Caucasian (100% vs 72%; p = 0.05), and more likely to be male (47.6% vs 39.0%; *p* = 0.4).

Approximately 11.1% and 12.5% of the otherwise asymptomatic women and men that respectively underwent DCMR experienced a total of 38 hospitalized clinical events over the average follow-up period of 58 months (Table 3). Of those with a myocardial infarction or unstable angina, all underwent a percutaneous coronary intervention except for 2 men with unstable angina who underwent coronary artery bypass grafting.

**Table 3:** Cardiovascular Events by Gender

	Women	Men
Death	10	6
Myocardial Infarction	3	2
Incident Heart Failure Warranting Hospitalization	3	1
Unstable Angina	2	5
Transient Ischemic Attack/Cerebrovascular Accident	4	2
None	177	112

List of cardiovascular events and death stratified by gender

In univariable analysis (Table 4) with both genders combined, SBP and age were associated with hospitalized CV events and survival, *p* = 0.002 and 0.01, respectively. For both genders combined, stress-induced LV wall motion abnormality was associated with hospitalized CV events and survival (*p* = 0.003). In those who received gadolinium contrast, the presence of a stress-induced perfusion defect was also associated with hospitalized CV events and survival (*p* = 0.007). When combining either a stress-induced perfusion defect or a DCMR-induced LV wall motion abnormality as evidence of myocardial ischemia, it is significantly associated with CV events and survival (*p* < 0.001). These associations appeared stronger in men than women, but the interaction term was not significant (*p* > 0.20).

**Table 4:** Univariate Cox Proportional Hazard Ratios for Cardiovascular Events/Survival

	All		Women		Men	
	HR (95% CI)	*p*-value	HR (95% CI)	*p*-value	HR (95% CI)	*p*-value
Age	1.05 (1.01–1.09)	0.01	1.05 (1.00–1.11)	0.04	1.04 (0.99–1.11)	0.14
Body Mass Index	1.00 (0.95–1.05)	0.86	1.00 (0.94–1.07)	0.91	1.01 (0.91–1.10)	0.82
Total Cholesterol	1.00 (0.99–1.01)	0.53	1.00 (0.99–1.01)	0.62	1.01 (0.98–1.02)	0.52
High Density Lipoprotein	0.99 (0.95–1.02)	0.59	0.98 (0.94–1.03)	0.55	0.99 (0.92–1.04)	0.84
Systolic Blood Pressure	1.03 (1.01–1.06)	0.002	1.03 (0.99–1.06)	0.08	1.04 (1.01–1.07)	0.007
Current Smoker	2.18 (0.53–6.09)	0.19	2.45 (0.39–8.46)	0.22	1.83 (0.10–9.13)	0.59
Diabetes Mellitus	1.3 (0.69–2.48)	0.40	2.82 (0.85–10.77)	0.11	0.95 (0.18–4.33)	0.52
Inducible Wall Motion Abnormality	3.30 (1.56–6.49)	0.003	0.88 (0.14–3.02)	0.86	8.86 (3.28–224.90)	< 0.001
Stress-Induced Perfusion Defect	2.79 (1.33–5.82)	0.007	1.62 (0.55–4.38)	0.36	5.55 (1.85–18.41)	0.003
Any Ischemia	3.13 (1.64–5.93)	< 0.001	1.63 (0.62–3.89)	0.30	7.41 (2.69–23.54)	< 0.001
Left Ventricular Ejection Fraction	0.99 (0.95–1.03)	0.65	1.04 (0.97–1.12)	0.21	0.96 (0.89–1.07)	0.06
Late Gadolinium Enhancement	2.41 (0.95–5.34)	0.06	0.71 (0.04–3.48)	0.72	4.39 (1.41–13.28)	0.01

Results of univariate Cox proportional hazard relationships between different risk factors and cardiovascular events/survival. Overall study population results included and those stratified by gender. Of note, for perfusion defects and late gadolinium enhancement, the study population consisted only of those 222 who received contrast

**Fig. 3:**
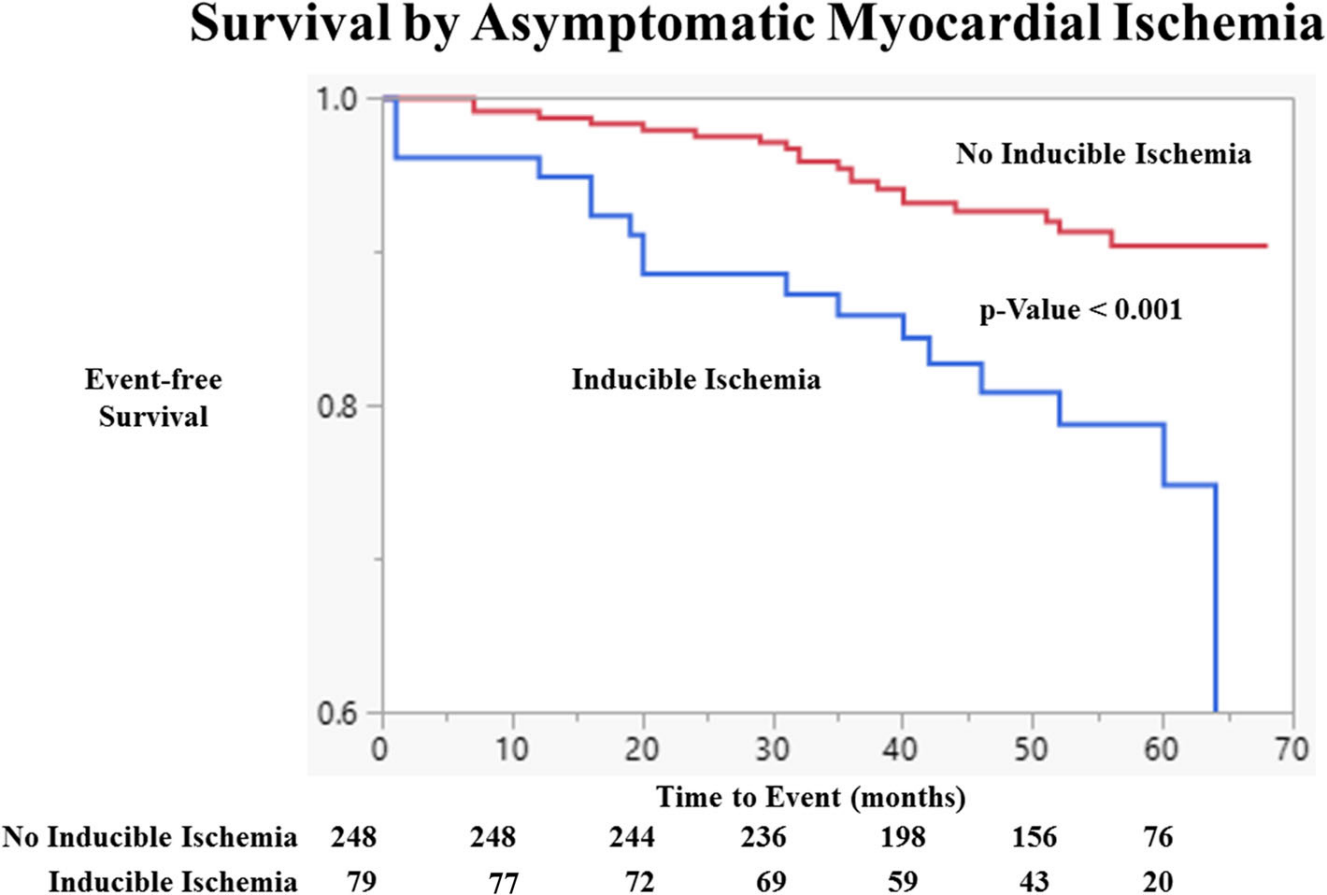
Event-free Survival by Asymptomatic Myocardial Ischemia. Kaplan Meier curves of cardiovascular event free as a function of length of follow-up for those with and without myocardial ischemia for the study population without known coronary artery disease (CAD). Test comparing the two groups is based on the log-rank test

**Fig. 4:**
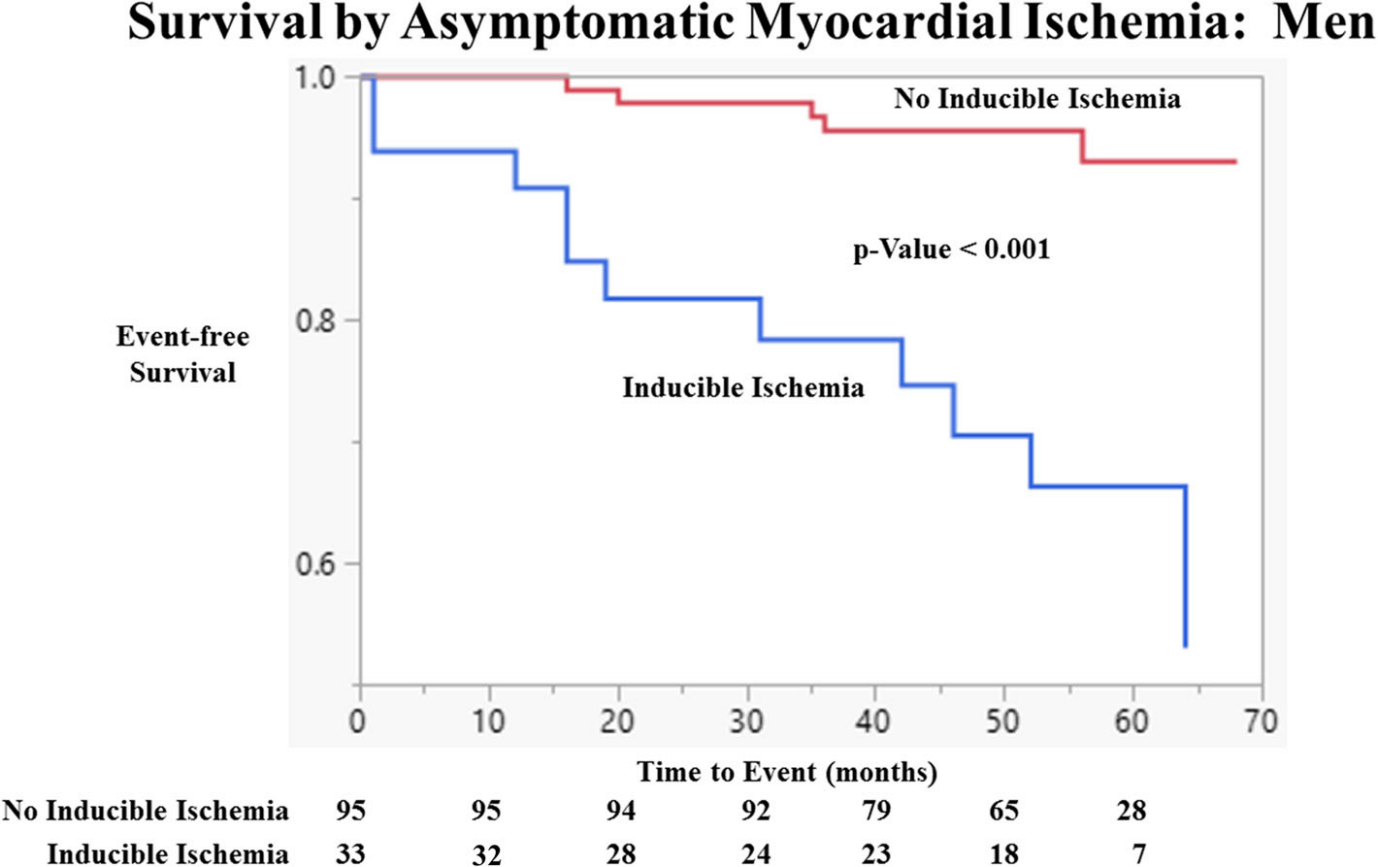
Event-free Survival by Asymptomatic Myocardial Ischemia: Men. Kaplan Meier curves of cardiovascular event free as a function of length of follow-up for men with and without myocardial ischemia on DCMR for the study population without known coronary artery disease. Test comparing the two groups is based on the log-rank test

**Fig. 5:**
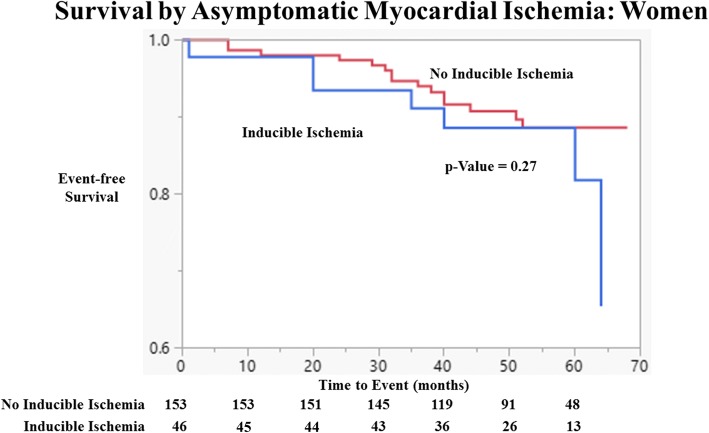
Cardiovascular Event-free Survival by Asymptomatic Myocardial Ischemia: Women. Kaplan Meier curves of cardiovascular event free as a function of length of follow-up for women with and without myocardial ischemia for the study population without known coronary artery disease. Test comparing the two groups is based on the log-rank test

DCMR measures of myocardial ischemia did improve the prediction of hospitalized CV events and survival overall. The composite event rate for hospitalized CV events or death was 8.0% and 22.8% for those without and with inducible myocardial ischemia (*p* < 0.001). In women, the composite event rates were 9.8% and 15.2% (*p* = 0.32), but in men, they were 5.3% and 33.3% (*p* < 0.001) for those without versus with inducible myocardial ischemia, respectively. In Kaplan-Meier analyses, myocardial ischemia was associated with a reduced event-free survival (*p* < 0.001). This pattern was seen more significantly in men compared to women (*p* < 0.001 and *p* = 0.27, respectively; see Figs. 3, 4 and 5).

To guard against compromising our results due to over-fitting our statistical models, we first performed multivariable stepwise Cox regression analysis in which the most significant contributors to events were compared with one another. As shown in Table 5, stress-induced myocardial ischemia predicted CV events/survival (*p* < 0.001) as well as tobacco use (*p* = 0.01). Other variables, such as SBP, diabetes mellitus, and LV mass, met model inclusion but did not reach statistical significance. Secondly, we performed additional Cox proportional hazard models for determining a participant’s HR of experiencing a hospitalized CV event/survival utilizing incremental adjustment models as detailed above. The crude HR for a hospitalized CV event/survival whether myocardial ischemia was present was 3.13 (95% CI: 1.64–5.93; *p* < 0.001; see Table 6). The significance persisted after adjustment for baseline demographics (*p* < 0.001) and after further adjustment for significant cardiovascular risk factors (*p* = < 0.001). Finally, after adjustment for imaging findings, such as LV ejection fraction and mass, myocardial ischemia continued to be associated with CV events and survival [HR: 4.07 (95% CI: 1.95–873); *p* < 0.001). To evaluate the fit of these models, the receiver operating curve was used to calculate the area under the curve, which was 0.710, 0.848, and 0.860 for models 1–3, respectively.

**Table 5:** Stepwise Multivariate Regression for Cardiovascular Events and Survival

	*p*-Value
LV Mass	0.051
Diabetes Mellitus	0.202
Current Tobacco Use	0.011
Systolic Blood Pressure	0.128
Asymptomatic Ischemia	< 0.001

Results of stepwise logistical regression evaluating multivariate association between variables and cardiovascular events/survival. In order to be included in the model, the p-value had to be less than 0.25

**Table 6:** Crude and Multivariate Cox Proportional Hazard Models of Cardiovascular Events/Survival by Myocardial Ischemia

	HR (95% CI)	*p*-Value
Unadjusted	3.13 (1.64–5.93)	< 0.001
Model 1	3.12 (1.62–5.97)	< 0.001
Model 2	4.42 (2.12–9.43)	< 0.001
Model 3	4.07 (1.95–8.73)	< 0.001

Results of incremental adjustment with Cox proportional hazard model. The hazard ratio is for myocardial ischemia and its relationship to cardiovascular events and survival

## Discussion

There are several important findings in this study. First, nearly a quarter of middle and older aged asymptomatic individuals with CV disease risk factors exhibited DCMR evidence of inducible “silent” myocardial ischemia (Table 2). Second, when asymptomatic myocardial ischemia was present, we observed men to experience more hospitalizations for a CV event or death than women. Finally, the presence of LV myocardial ischemia in this asymptomatic population was most predictive of a future hospitalized CV event if they had no prior CV event and no known history of CAD – both conditions for which current algorithms and appropriate use guidelines do not recommend stress testing.

As shown in Table 3, we observed a total of 38 hospitalized CV events or deaths (event rate of 11.6% over 5 years) which was lower than what we might have extrapolated from other studies such as the Framingham Heart Study cohort [15]. This may have been due to: a) a United States nationwide decline in the incidence of hospitalized CV events since publication of the initial Framingham Heart Study data [16], b) the majority of the participants (67%) received HMG Co-A reductase inhibitors (i.e. statin medications) which have been shown to reduce the incidence of hospitalized CV events [17], c) people who volunteer for studies tend to be healthier and less likely to develop a CV event warranting hospitalization when compared to non-responders [18], and d) the participants in the study were contacted by the research nurse every 4 months; such close follow-up could have changed their behavior leading to better compliance with medical or behavioral treatment directed toward reducing the risk of a hospitalization for a CV event [20].

Silent myocardial ischemia has even been investigated in different stress testing modalities. In high-risk patients with type 2 diabetes mellitus for at least 15 years, the positive predictive value for silent myocardial ischemia by dobutamine stress echocardiography was 69%, 75% for single photon emission computed tomography (SPECT), and 60% for exercise stress testing [26]. While silent myocardial ischemia remains an elusive diagnosis, most clinicians more readily appreciate silent myocardial infarctions. The three risk factors most commonly associated with silent myocardial infarctions are diabetes mellitus, hypertension, and advanced age [27–30]. In our study, most individuals exhibited hypertension. This study demonstrated that DCMR in otherwise asymptomatic middle aged and older individuals identifies silent myocardial ischemia which forecasted CV events.

As one might expect in a multivariable analysis, current smoking and myocardial ischemia were associated with future cardiac events (Table 5). The unexpected finding in this study relates to the fact that the association between DCMR induced myocardial ischemia and hospitalized CV events/survival was driven by the strong association of stress induced LV wall motion abnormality or perfusion defects in men—a population that otherwise would not undergo pharmacologic stress testing.

LV myocardial ischemia had a weaker ability to identify subsequent CV events in women. There are several potential reasons for this. First, in a prior study (24), gender-related differences in sensitivity for diagnosing CAD in women was partially attributed to women achieving target heart rates at lower dobutamine doses with less frequent use of atropine. In the current study, compared to men, women achieved their target heart rate at lower dobutamine doses (*p* = 0.004), and the total atropine dose was lower in women (*p* = 0.05, Table 2). Second, within the same age range, women experienced fewer hospitalized CV events than men. They experienced more deaths and cerebrovascular events than men (Table 3) for which the stress CMR may not have readily identified. It is possible that with a larger sample size that included older women (and thus an increased likelihood of experiencing a hospitalization for a CV event), we would be able to improve prediction of hospitalized CV events in women.

>While the results of this study remain intriguing, few data are available to direct our diagnostic and therapeutic approach for at-risk but asymptomatic middle aged and older individuals at risk for a future CV event. Most of the guideline-based approaches focus on symptomatic individuals and lack clarity in how to approach patients who are asymptomatic [10, 12, 14, 15, 31, 32]. Under most circumstances, stress tests are not indicated unless a patient has symptoms suggestive of a coronary etiology. The results of this study suggest future research is necessary to develop evidence-based strategies for clinicians to determine how and when to identify and potentially treat asymptomatic middle aged and older men at risk for future CV events.

There are limitations pertaining to this study. First, this study actively recruited individuals with known long-standing hypertension or diabetes mellitus. As a result, our findings mainly relate to persons with long-term CV risk factor exposure, and in these particular analyses, since participants with known CAD were excluded, the individuals remaining with significant risk factors may be more resistant to developing clinically-significant CAD. Second, the true burden of silent myocardial ischemia remains unknown given the high-risk populations which were enrolled in this study. More inclusive studies would need to better define the risk of those who are at low- or intermediate-level risk. Third, there were fewer events than forecasted for the initial sample size estimates for this study. At the time of study inception, using published data, the hospitalized CV event rate was forecasted to be 4% to 7% per year. The lower than anticipated hospitalized cardiac event rate means that larger studies are needed to examine the impact of a multiplicity of risk factors toward promoting CV events. The power of our study is estimated to be 0.6. To capture sufficient events to perform more meaningful analyses would require a sample size of 2000–5000 individuals depending on the event rates used. Finally, since only 68% of the 327 participants received CMR gadolinium contrast to assess first pass perfusion, there is a risk that asymptomatic stress-induced perfusion defects are under-reported.

## Conclusion

Among asymptomatic middle-aged individuals with risk factors for a sentinel CV event, the presence of myocardial ischemia during DCMR forecasted a future hospitalized CV event or death. Further studies are needed in middle aged and older individuals to more accurately characterize the prevalence, significance, and management of asymptomatic myocardial ischemia.

## Abbreviations

ACC: American College of Cardiology
AHA: American Heart Association
CAD: Coronary artery disease
CI: Confidence interval
CMR: Cardiovascular magnetic resonance
CV: Cardiovascular
DBP: Diastolic blood pressure
DCMR: Dobutamine stress cardiovascular magnetic resonance
ESC: European Society of Cardiology
FA: Flip angle
FOV: Field of view
HR: Hazard ratio
LV: Left ventricle/left ventricular
MI: Myocardial infarction
SBP: Systolic blood pressure
SPECT: Single photon emission computed tomography
TE: Echo time
TR: Repetition time
